# Supplementary material for: Baby skyrmions in Chern ferromagnets and topological mechanism for spin-polaron formation in twisted bilayer graphene
Source: Nat Commun. 2022 Oct 21;13:6245. doi: 10.1038/s41467-022-33673-3 (PMC9587044; doi:10.1038/s41467-022-33673-3)
Supplement: Supplementary file 1 — Supplementary Information [file 41467_2022_33673_MOESM1_ESM.pdf]

Supplementary Information

Baby skyrmions in Chern ferromagnets and topological mechanism for spin-polaron formation in twisted bilayer graphene

Khalaf and Vishwanath

In this supplementary mateiral, we consider in some detail the formation of the spin polaron bound state in the LLL. The problem simplifies in this case due to contineous magnetic translation symmetry. The form factors for the LLL are given by

$$\lambda_{\boldsymbol{q}}(\boldsymbol{k}) = \langle u_{\boldsymbol{k}} | u_{\boldsymbol{k}+\boldsymbol{q}} \rangle = e^{-\frac{|B|}{4} q^2 - i \frac{B}{2} \boldsymbol{q} \wedge \boldsymbol{k}} \tag{1}$$

where as in the main text, we take a unit cell which encloses one magnetic flux such that $|B| = \frac{2\pi}{A_{\rm BZ}}$. We can now use the properties of the GMP algebra [1] to understand the formation the bound states.

In the following, we will follow the notation of Ref. [2] where $\hat{A} = \sum_{\alpha,\beta} c_\alpha^\dagger A_{\alpha\beta} c_\beta$ and introducing the matrix $[\lambda_q]_{\boldsymbol{k},\boldsymbol{k}'} = \lambda_{\boldsymbol{q}}(\boldsymbol{k}) \delta_{\boldsymbol{k}',\boldsymbol{k}+\boldsymbol{q}}$ such that $\rho_{\boldsymbol{q}} = \hat{\lambda}_{\boldsymbol{q}}$. We will also use the relation [2]

$$[\hat{A}, \hat{B}] = \widehat{[A, B]} \tag{2}$$

We can see that the GMP algebra [1] follows from the matrix commutation relation

$$\begin{aligned}
[\lambda_{\boldsymbol{q}}, \lambda_{\boldsymbol{q}'}]_{\boldsymbol{k},\boldsymbol{k}'} &= \delta_{\boldsymbol{k}',\boldsymbol{k}+\boldsymbol{q}+\boldsymbol{q}'} [\lambda_{\boldsymbol{q}}(\boldsymbol{k}) \lambda_{\boldsymbol{q}'}(\boldsymbol{k}+\boldsymbol{q}) - \lambda_{\boldsymbol{q}'}(\boldsymbol{k}) \lambda_{\boldsymbol{q}}(\boldsymbol{k}+\boldsymbol{q}')] = 2i \sin \frac{B}{2} \boldsymbol{q} \wedge \boldsymbol{q}' \, e^{-\frac{B}{4}(\boldsymbol{q}^2 + \boldsymbol{q}'^2) - i\frac{B}{2}(\boldsymbol{q}+\boldsymbol{q}') \wedge \boldsymbol{k}} \delta_{\boldsymbol{k}',\boldsymbol{k}+\boldsymbol{q}+\boldsymbol{q}'} \\
&= 2i \sin\left(\frac{B}{2} \boldsymbol{q} \wedge \boldsymbol{q}'\right) e^{\frac{B}{2} \boldsymbol{q} \cdot \boldsymbol{q}'} [\lambda_{\boldsymbol{q}+\boldsymbol{q}'}]_{\boldsymbol{k},\boldsymbol{k}'}
\end{aligned} \tag{3}$$

For this choice of of form factors, we need to make a specific gauge choice for the operators $c_{\boldsymbol{k}}$ such that $\rho_{\boldsymbol{q}}$ does not change under shifting $\boldsymbol{k}$ in the summation by a reciprocal lattice vector $\boldsymbol{G}$. This is realized by taking

$$c_{\boldsymbol{k}} = c_{[\boldsymbol{k}]} e^{i\phi_{\boldsymbol{k}}}, \qquad \phi_{\boldsymbol{k}} = -\frac{B}{2} \{\boldsymbol{k}\} \wedge [\boldsymbol{k}] - \frac{B}{4} \{\boldsymbol{k}\} \wedge \{\bar{\boldsymbol{k}}\} \tag{4}$$

where we decomposed $\boldsymbol{k}$ into $[\boldsymbol{k}]$ which lies in the first BZ and $\{\boldsymbol{k}\} = \boldsymbol{k} - [\boldsymbol{k}]$ which is a reciprocal lattice vector. We also introduced the bar notation such that for a vector $\boldsymbol{k} = k_1 \boldsymbol{b}_1 + k_2 \boldsymbol{b}_2$, where $\boldsymbol{b}_1$ are the momentum space basis vectors, $\bar{\boldsymbol{k}} = k_1 \boldsymbol{b}_1 - k_2 \boldsymbol{b}_2$. To see how this makes $\rho_{\boldsymbol{q}}$ invariant under shifts in $\boldsymbol{k}$, we note the following identities. Assuming $\boldsymbol{b}_1 \wedge \boldsymbol{b}_2 = +A_{\rm BZ}$, we can write

$$\boldsymbol{n} = n_1 \boldsymbol{b}_1 + b_2 \boldsymbol{b}_2, \quad \boldsymbol{m} = m_1 \boldsymbol{b}_1 + m_2 \boldsymbol{b}_2, \tag{5}$$

$$\boldsymbol{n} \wedge \bar{\boldsymbol{m}} = -A_{\rm BZ}(n_1 m_2 + n_2 m_2) = \boldsymbol{m} \wedge \bar{\boldsymbol{n}}, \qquad \boldsymbol{n} \wedge \bar{\boldsymbol{n}} = -2A_{\rm BZ} n_2 n_2 \tag{6}$$

Thus, we get

$$c_{\boldsymbol{k}+\boldsymbol{G}} = c_{\boldsymbol{k}} e^{-i\frac{B}{2} \boldsymbol{G} \wedge [\boldsymbol{k}] - i\frac{B}{2} \{\boldsymbol{k}\} \wedge \bar{\boldsymbol{G}} - i\frac{B}{4} \boldsymbol{G} \wedge \bar{\boldsymbol{G}}}, \tag{7}$$

which yields

$$\begin{aligned}
c_{\boldsymbol{k}+\boldsymbol{G}}^\dagger c_{\boldsymbol{k}+\boldsymbol{q}+\boldsymbol{G}} \lambda_{\boldsymbol{q}}(\boldsymbol{k}+\boldsymbol{G}) &= c_{\boldsymbol{k}}^\dagger c_{\boldsymbol{k}+\boldsymbol{q}} \lambda_{\boldsymbol{q}}(\boldsymbol{k}) e^{i\frac{B}{2} \boldsymbol{G} \wedge [\boldsymbol{k}] + i\frac{B}{2} \{\boldsymbol{k}\} \wedge \bar{\boldsymbol{G}}} e^{-i\frac{B}{2} \boldsymbol{G} \wedge [\boldsymbol{k}+\boldsymbol{q}] - i\frac{B}{2} \{\boldsymbol{k}+\boldsymbol{q}\} \wedge \bar{\boldsymbol{G}}} e^{-i\frac{B}{2} \boldsymbol{q} \wedge \boldsymbol{G}} \\
&= c_{\boldsymbol{k}}^\dagger c_{\boldsymbol{k}+\boldsymbol{q}} \lambda_{\boldsymbol{q}}(\boldsymbol{k}) e^{-i\frac{B}{2} \{\boldsymbol{k}+\boldsymbol{q}\} \wedge (\boldsymbol{G}+\bar{\boldsymbol{G}})} = c_{\boldsymbol{k}}^\dagger c_{\boldsymbol{k}+\boldsymbol{q}} \lambda_{\boldsymbol{q}}(\boldsymbol{k}) e^{-2\pi i C \{\boldsymbol{k}+\boldsymbol{q}\}_2 G_1} = c_{\boldsymbol{k}}^\dagger c_{\boldsymbol{k}+\boldsymbol{q}} \lambda_{\boldsymbol{q}}(\boldsymbol{k})
\end{aligned} \tag{8}$$

On going from the first to the second line, we have assumed $\boldsymbol{k}$ lies in the first BZ such that $\{\boldsymbol{k}\} = 0$. In the second inequality we wrote the $\boldsymbol{G} = G_1 \boldsymbol{b}_1 + G_2 \boldsymbol{b}_2$ and $\{\boldsymbol{k}+\boldsymbol{q}\} = \{\boldsymbol{k}+\boldsymbol{q}\}_1 \boldsymbol{b}_1 + \{\boldsymbol{k}+\boldsymbol{q}\}_2 \boldsymbol{b}_2$ and used $B\boldsymbol{b}_1 \wedge \boldsymbol{b}_2 = -BA_{\rm BZ} = -2\pi$. Finally, we used the fact that $\{\boldsymbol{k}+\boldsymbol{q}\}_{1,2}$ and $G_{1,2}$ are integers.

Let us define $\tilde{\rho}_{\boldsymbol{q}} = \rho_{\boldsymbol{q}} e^{\frac{|B|}{4} \boldsymbol{q}^2}$ which satisfy the modified GMP algebra

$$[\tilde{\rho}_{\boldsymbol{q}}, \tilde{\rho}_{\boldsymbol{q}'}] = 2i \sin\left(\frac{B}{2} \boldsymbol{q} \wedge \boldsymbol{q}'\right) \tilde{\rho}_{\boldsymbol{q}+\boldsymbol{q}'} \tag{9}$$

With this definition, we find that

$$\tilde{\rho}_{\boldsymbol{q}} = \sum_{\sigma,\boldsymbol{k}\in{\rm BZ}} c_{\sigma,\boldsymbol{k}}^\dagger c_{\sigma,[\boldsymbol{k}+\boldsymbol{q}]} \tilde{\lambda}_{\boldsymbol{q}}(\boldsymbol{k}) = \sum_{\sigma,\boldsymbol{k}\in{\rm BZ}} \tilde{c}_{\sigma,\boldsymbol{k}}^\dagger \tilde{c}_{\sigma,\boldsymbol{k}+\boldsymbol{q}} \tilde{\lambda}_{\boldsymbol{q}}(\boldsymbol{k}), \qquad \tilde{\lambda}_{\boldsymbol{q}}(\boldsymbol{k}) = e^{-i\frac{B}{2} \boldsymbol{q} \wedge \boldsymbol{k}} e^{i(\phi_{\boldsymbol{k}+\boldsymbol{q}} - \phi_{\boldsymbol{k}})} \tag{10}$$

In the following, we will assume that the gauge is periodic such that $c_{\boldsymbol{k}} = c_{[\boldsymbol{k}]}$.

The spin waves in the model can be understood by defining the spin raising operator

$$\tilde{S}^+_{\boldsymbol{q}} = \frac{1}{\sqrt{N}} \sum_{\boldsymbol{k}} c^\dagger_{\uparrow,\boldsymbol{k}} c_{\downarrow,\boldsymbol{k}+\boldsymbol{q}} \tilde{\lambda}_{\boldsymbol{q}}(\boldsymbol{k}) = \widehat{\tilde{\lambda}_{\boldsymbol{q}} \otimes \sigma^+}, \qquad \sigma^+ = \frac{1}{2}(\sigma_x + i\sigma_y) \tag{11}$$

It is easy to verify that the commutation relation

$$[\delta\tilde{\rho}_{\boldsymbol{q}}, \tilde{S}^+_{\boldsymbol{q}'}] = 2i \sin\left(\frac{B}{2}\boldsymbol{q} \wedge \boldsymbol{q}'\right) \tilde{S}^+_{\boldsymbol{q}+\boldsymbol{q}'} \tag{12}$$

which implies that $|\boldsymbol{q}\rangle = \tilde{S}^+_{\boldsymbol{q}} |\downarrow\rangle$ is an exact eigenstate of $\mathcal{H}$ (note that for the LLL, the single particle term $\epsilon_0(\boldsymbol{k})$ vanishes) since

$$\mathcal{H}|\boldsymbol{q}\rangle = \xi_{\boldsymbol{q}}|\boldsymbol{q}\rangle, \qquad \xi_{\boldsymbol{q}'} = \frac{2}{A}\sum_{\boldsymbol{q}'} \tilde{V}_{\boldsymbol{q}'} \sin^2\left(\frac{B}{2}\boldsymbol{q}\wedge\boldsymbol{q}'\right) \simeq 2\rho \boldsymbol{q}^2 + O(\boldsymbol{q}^4), \qquad \rho = \frac{B^2}{8A}\sum_{\boldsymbol{q}'} \tilde{V}_{\boldsymbol{q}'} {\boldsymbol{q}'}^2 = \frac{B^2}{16\pi}\int dq q^3 V_q e^{-\frac{B}{2}q^2} \tag{13}$$

In addition $|\boldsymbol{q}\rangle$ satisfy

$$\langle \boldsymbol{q}'|\boldsymbol{q}\rangle = \frac{1}{N}\sum_{\boldsymbol{k},\boldsymbol{G}} \delta_{\boldsymbol{q},\boldsymbol{q}'+\boldsymbol{G}} \tilde{\lambda}^*_{\boldsymbol{q}'}(\boldsymbol{k})\tilde{\lambda}_{\boldsymbol{q}}(\boldsymbol{k}) = \frac{1}{N}\sum_{\boldsymbol{G}} \delta_{\boldsymbol{q},\boldsymbol{q}'+\boldsymbol{G}} e^{-i\frac{B}{2}\boldsymbol{G}\wedge\boldsymbol{q} - i\frac{B}{2}\boldsymbol{G}\wedge\bar{\boldsymbol{G}}} \sum_{\boldsymbol{k}} e^{-iB\boldsymbol{G}\wedge\boldsymbol{k}} = \delta_{\boldsymbol{q},\boldsymbol{q}'} \tag{14}$$

where $\boldsymbol{q}$ ranges over the linearly indepdent generators of the GMP algebra given by $\boldsymbol{q} = q_1\boldsymbol{b}_1 + q_2\boldsymbol{b}_2$, $0 \leq q_1 < N_1$, $0 \leq q_2 < N_2$. Note that this is different from the case of a general Chern band where $\boldsymbol{q}$ is defined to live only within the first BZ with an additional index n that goes from 1 to N. Here instead $\boldsymbol{q}$ itself ranges over N^2 points in the extended BZ.

We define the states

$$|\boldsymbol{k}_0; \boldsymbol{q}\rangle = \tilde{\lambda}^*_{\boldsymbol{q}}(\boldsymbol{k}_0) c^\dagger_{\uparrow,\boldsymbol{k}_0+\boldsymbol{q}} \tilde{S}^+_{\boldsymbol{q}} |\downarrow\rangle \tag{15}$$

which satisfy

$$\langle \boldsymbol{k}'_0; \boldsymbol{q}'|\boldsymbol{k}_0; \boldsymbol{q}\rangle = \frac{1}{N}\tilde{\lambda}_{\boldsymbol{q}'}(\boldsymbol{k}'_0)\tilde{\lambda}^*_{\boldsymbol{q}}(\boldsymbol{k}_0)\sum_{\boldsymbol{k},\boldsymbol{k}'} \tilde{\lambda}^*_{\boldsymbol{q}'}(\boldsymbol{k}')\lambda_{\boldsymbol{q}}(\boldsymbol{k})\langle c^\dagger_{\downarrow,\boldsymbol{k}'+\boldsymbol{q}'} c_{\uparrow,\boldsymbol{k}'} c_{\uparrow,\boldsymbol{k}'_0+\boldsymbol{q}'} c^\dagger_{\uparrow,\boldsymbol{k}_0+\boldsymbol{q}} c^\dagger_{\uparrow,\boldsymbol{k}} c_{\downarrow,\boldsymbol{k}+\boldsymbol{q}}\rangle \tag{16}$$

$$= \frac{1}{N}\tilde{\lambda}_{\boldsymbol{q}'}(\boldsymbol{k}'_0)\tilde{\lambda}^*_{\boldsymbol{q}}(\boldsymbol{k}_0)\sum_{\boldsymbol{k},\boldsymbol{k}'} \tilde{\lambda}^*_{\boldsymbol{q}'}(\boldsymbol{k}')\lambda_{\boldsymbol{q}}(\boldsymbol{k})[\delta_{\boldsymbol{k}'+\boldsymbol{q}',\boldsymbol{k}+\boldsymbol{q}}\delta_{\boldsymbol{k},\boldsymbol{k}'}\delta_{\boldsymbol{k}'_0+\boldsymbol{q}',\boldsymbol{k}_0+\boldsymbol{q}} - \delta_{\boldsymbol{k}'+\boldsymbol{q}',\boldsymbol{k}+\boldsymbol{q}}\delta_{\boldsymbol{k},\boldsymbol{k}'_0+\boldsymbol{q}'}\delta_{\boldsymbol{k}',\boldsymbol{k}_0+\boldsymbol{q}}] \tag{17}$$

$$= \delta_{\boldsymbol{k}_0,\boldsymbol{k}'_0}[\delta_{\boldsymbol{q},\boldsymbol{q}'} - \frac{1}{N}\tilde{\lambda}_{\boldsymbol{q}'}(\boldsymbol{k}_0)\tilde{\lambda}^*_{\boldsymbol{q}}(\boldsymbol{k}_0)\tilde{\lambda}^*_{\boldsymbol{q}'}(\boldsymbol{k}_0+\boldsymbol{q})\tilde{\lambda}_{\boldsymbol{q}}(\boldsymbol{k}_0+\boldsymbol{q}')] \tag{18}$$

where we assumed that $\boldsymbol{k}_0$ and $\boldsymbol{k}'_0$ lie in the first BZ. The action of $\mathcal{H}$ on $|\boldsymbol{k},\boldsymbol{q}\rangle$ is given by

$$\mathcal{H}|\boldsymbol{k}_0;\boldsymbol{q}\rangle = \xi_{\boldsymbol{q}}|\boldsymbol{k}_0;\boldsymbol{q}\rangle + \frac{i}{A}\sum_{\boldsymbol{q}'}\tilde{V}_{\boldsymbol{q}'}\sin\left(\frac{B}{2}\boldsymbol{q}'\wedge\boldsymbol{q}\right)(e^{i\frac{B}{2}\boldsymbol{q}'\wedge\boldsymbol{q}}|\boldsymbol{k}_0;\boldsymbol{q}+\boldsymbol{q}'\rangle - e^{-i\frac{B}{2}\boldsymbol{q}'\wedge\boldsymbol{q}}|\boldsymbol{k}_0;\boldsymbol{q}-\boldsymbol{q}'\rangle) \tag{19}$$

As we can see, the Hamiltonian is independent on $\boldsymbol{k}_0$ which implies that all bands, single particle and polaron, are perfectly flat. This is a consequence of contineous magnetic translation.

The bound state wavefunction can be written as

$$|\Psi\rangle = \sum_{\boldsymbol{q}} r_{\boldsymbol{q}}|\boldsymbol{k}_0;\boldsymbol{q}\rangle \tag{20}$$

We reiterate here that $\boldsymbol{q}$ is not restricted to the first BZ. The magnitude and phase of corresponding to the bound state are shown in supplementary Fig. 1 (this corresponds to unfolding the wavefunction in Fig. 2 in the main text). We can see that the function decays exponentially away from $\boldsymbol{q}$ with its phase winding by 2π around $\boldsymbol{q} = 0$. This leads us to propose the variational ansatz

$$r_{\boldsymbol{q}}(\xi) = e^{-\frac{\xi}{2}|\boldsymbol{q}| + i\phi_{\boldsymbol{q}}}, \quad \phi_{\boldsymbol{q}} = \arg(q_x + iq_y) \qquad |\psi_\xi\rangle = \sum_{\boldsymbol{q}} r_{\boldsymbol{q}}(\xi)|\boldsymbol{q},\boldsymbol{q}\rangle \tag{21}$$

The overlap of the ansatz wavefunction with the exact ground state as a function of ξ (measured in units of l_B) is shown in supplementary Fig. 1 and we can see that for the optimal value of ξ around $\xi \approx 2.6$, we get an overlap exceeding 99%. We can also see in supplementary Fig. 1, the minimum variational energy compared to the exact ground state energy and we see the two match very closely.

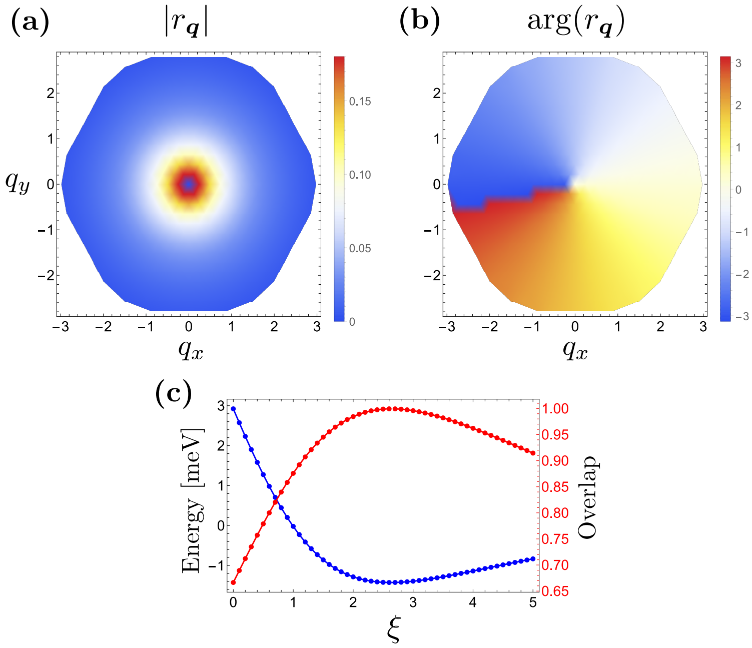

Supplementary Figure 1: **(a)** Mangitude of the bound state wavefunction, **(b)** Phase of the bound state wavefunction, and **(c)** Variational energy and overlap with the exact wavefunction as a function of the variational parameter ξ measured in units of l_B.

Supplementary References

[1] S. M. Girvin, A. H. MacDonald, and P. M. Platzman. Magneto-roton theory of collective excitations in the fractional quantum hall effect. *Phys. Rev. B*, 33:2481–2494, Feb 1986.

[2] Eslam Khalaf, Shubhayu Chatterjee, Nick Bultinck, Michael P. Zaletel, and Ashvin Vishwanath. Charged skyrmions and topological origin of superconductivity in magic-angle graphene. *Science Advances*, 7(19):eabf5299, 2021.
